# Supplementary material for: Observed variations in U.S. frost timing linked to atmospheric circulation patterns
Source: Nat Commun. 2017 May 23;8:15307. doi: 10.1038/ncomms15307 (PMC5457496; doi:10.1038/ncomms15307)
Supplement: Supplementary Information — Supplementary Figures and Supplementary Table [file ncomms15307-s1.pdf]

	Spring (March-May)				Fall (September-November)			
	North	Central	South	West	North	Central	South	West
	S_N[1]	S_C[2]	S_S[3]	S_W[4]	F_N[5]	F_C[6]	F_S[7]	F_W[8]
T_H[9]	**0.16***	**0.14***	**0.09***	**0.08***	**0.27**	0.01	**0.05**	**0.17**
T_R[10]	**0.06***	**0.06***	**0.27***	**0.44***	**0.16**	**0.16**	**0.20**	**0.26**
C_{XX}[11]	**0.32**	**0.26**	**0.38**	**0.48**	**0.41**	**0.25**	**0.38**	**0.29**
$T_{MAM,SON}$[12]	**0.12***	**0.09***	**0.14***	**0.06***	**0.25**	0.03	**0.05**	**0.20**
T_G[13]	**0.14***	**0.14***	**0.05***	**0.08***	**0.24**	0.00	**0.04**	**0.13**
**PNAI**[14]	0.01	0.01	**0.04**	**0.29***	**0.07***	0.00*	**0.18***	**0.10**
**NAOI**[15]	0.00*	0.00	0.01	0.00*	**0.04***	0.00	0.00	0.00*
**AMOI**[16]	0.00	0.01*	**0.04***	0.02*	**0.11**	**0.05**	0.01	**0.10**
**SOI**[17]	**0.06***	0.03*	0.00*	0.03	0.02	0.00*	0.03	0.00*

[1] day of year with first spring frost in the North region
[2] day of year with first spring frost in the Central region
[3] day of year with first spring frost in the South region
[4] day of year with first spring frost in the West region
[5] day of year with last fall frost in the North region
[6] day of year with last fall frost in the Central region
[7] day of year with last fall frost in the South region
[8] day of year with last fall frost in the West region
[9] Northern Hemisphere annual mean temperature
[10] regional mean temperature for spring or fall according to column heading
[11] circulation index corresponding to frost timing index in the column heading, meaning C_{SN} for S_N, C_{SC} for S_C, etc.
[12] Northern Hemisphere mean temperature for March-May (T_{MAM}) or September-November (T_{SON}) according to column heading
[13] global annual mean temperature
[14] Pacific North American Pattern Index for March-May or September-November according to column heading
[15] North Atlantic Oscillation Index for March-May or September-November according to column heading
[16] Atlantic Multidecadal Oscillation Index for March-May or September-November according to column heading
[17] Southern Oscillation Index for March-May or September-November according to column heading

Supplementary Table 1. Squared correlation between indices of frost timing, temperature, and circulation, with bold indicating statistical significance and asterisk indicating negative correlation.

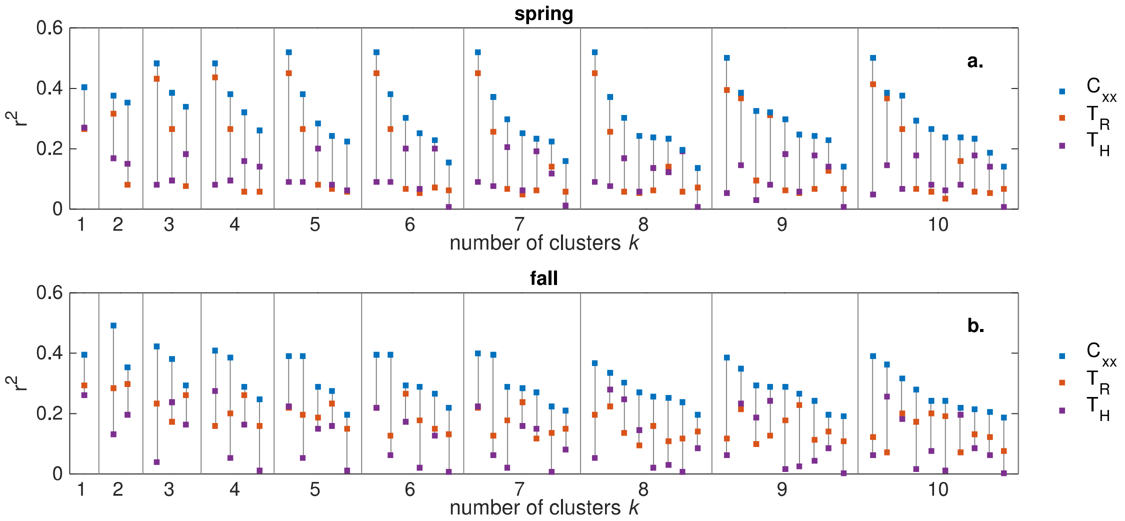

Supplementary Figure 1 | Robustness to number of clusters. (**a**) For values , the squared correlation between the day of year with last spring frost in each of the *k* regions and the following three time series: the circulation index for the region (C_{xx}), mean temperature in the region during spring (T_R), and hemispheric annual mean temperature (T_H). (**b**) Same as (**a**), but for the day of year with first fall frost.

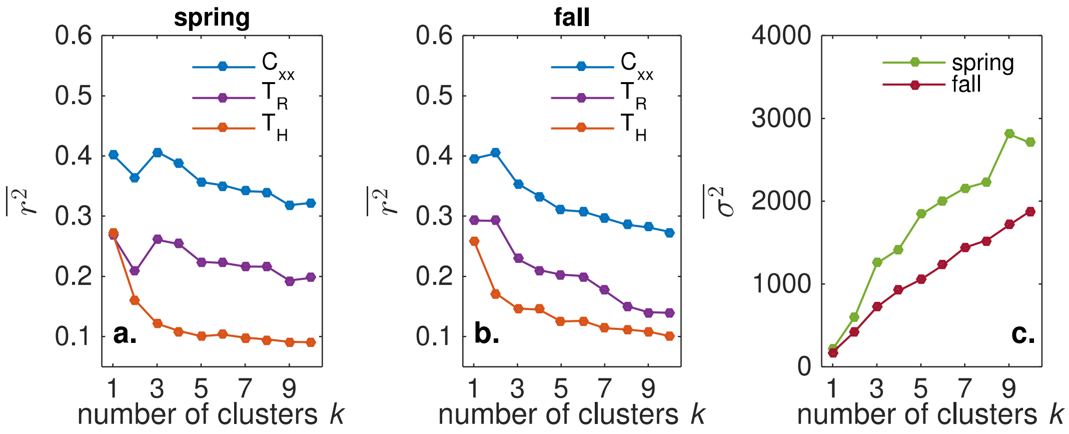

Supplementary Figure 2 | Variability versus number of clusters. (a) For number of clusters *k* in the range , the area-weighted mean squared correlation () between the day of year with last spring frost in each of the *k* regions and the following three time series: the circulation index specific to the region (C_{xx}), seasonal mean temperature in the region (T_R), and hemispheric annual mean temperature (T_H). **(b)** Same as **(a)**, but for the day of year with first fall frost. **(c)** The area-weighted mean variance of the day of year with last spring frost and first fall frost.

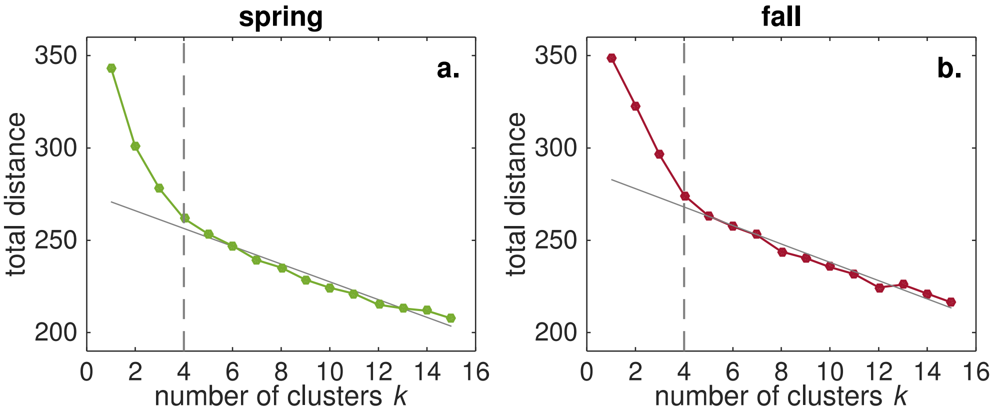

Supplementary Figure 3 | Distance versus number of clusters. (**a**) For clustering of the time series indicating day of year with last spring frost over the conterminous U.S., the curve indicates the total distance versus number of clusters *k*. Distance was defined as (1- R), where R is the correlation distance (Methods section). A least-sqaures fit to the values over is shown by the gray line, and the vertical line at 4 indicates the value of *k* used for the main results. (**b**) Same as (**a**), but for clustering of the first day of year with fall frost.

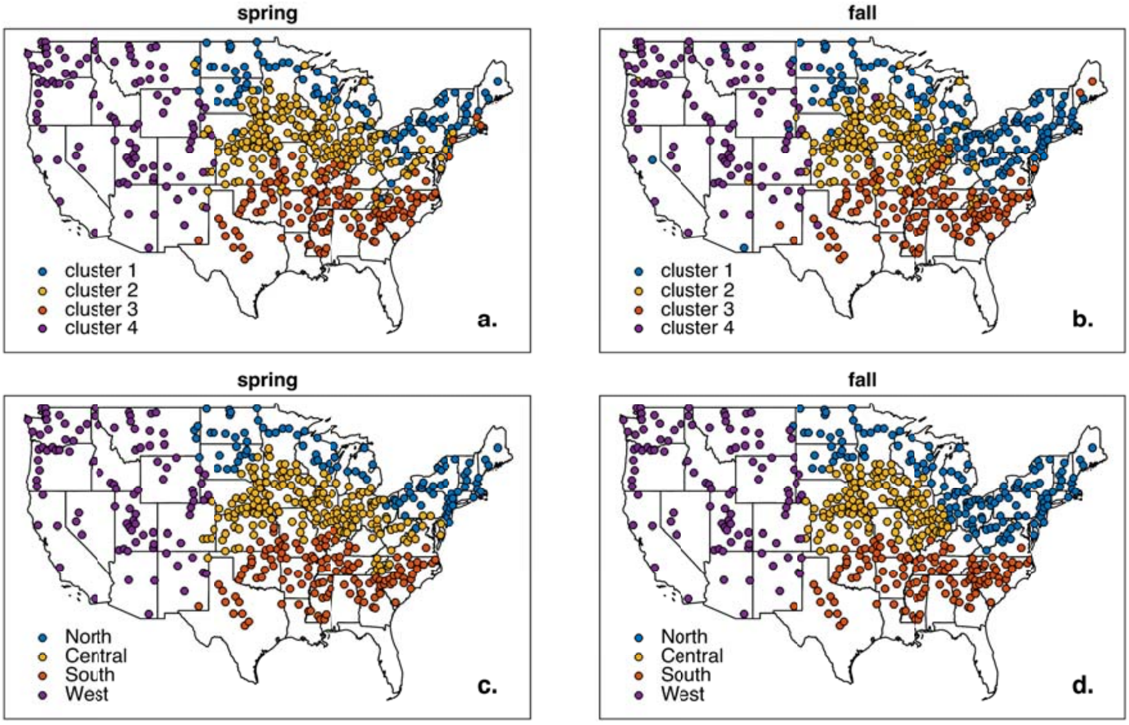

Supplementary Figure 4 | Objective clustering of stations. (a) Objective *k*-means clustering of the day of year with last spring frost (*k*=4; years 1920-2012). **(b)** Objective *k*-means clustering of the day of year with first fall frost (*k*=4; years 1920-2012). **(c)** As in (a), but outliers were eliminated by assignining each station the mode of the cluster assignments of its seven nearest neighbors. **(d)** As in (**b**), but outliers were eliminated by assignining each station the mode of the cluster assignments of its seven nearest neighbors.
